# Stigma and mental health in infectious diseases—meet the guest editors

**DOI:** 10.1186/s44263-024-00041-w

**Published:** 2024-02-12

**Authors:** Amrita Daftary, Jeremiah Chikovore

**Affiliations:** 1 School of Global Health, Dahdaleh Institute of Global Health Research, York University, Toronto, Canada; 2 Centre for the AIDS Programme of Research in South Africa, University of KwaZulu-Natal, Durban, South Africa; 3 Human Sciences Research Council, Durban, South Africa

In this Q&A, Amrita Daftary and Jeremiah Chikovore, guest editors for the journal’s collection on stigma and mental health in infectious diseases, reflect on their long-standing collaborative relationship and the substantial elements they have in common even while following unique pathways. They also share their perspectives regarding pressing issues in the field of stigma and mental health in infectious diseases, and how they see the field evolving.

## Q1. What is the focus of your research and what drew you to this field?

*Amrita Daftary*: I have always been both intrigued and enraged by avoidable injustices. Infectious diseases and the stigma associated with it are exemplary of this. My approach to research in this field has been informed by my training and experiences working with communities affected by TB and HIV in South Africa and India, among other settings. As a social and behavioral health scholar, I am most curious about the social dimensions of these illnesses. I am especially interested in those aspects that are less tangible, difficult to measure and therefore easier to miss. I enjoy using qualitative approaches in my research. Coupled with my natural inclination to fight injustice, I am especially drawn to critical research, and applying multiple methods to unearth nuances, question norms, and advocate for meaningful attention to the elements of care that are at times neglected in our quest for disease eradication and infection control, yet which can matter gravely to the overall health and wellbeing of those who become affected. I sit in Canada, a country with a mired history of TB and HIV yet in many ways once-removed from the more extensive epidemics ravaging the global south, a part of the world where I too once lived. My continuing work in TB and HIV continues to be shaped by this discord in close allyship with affected communities.

*Jeremiah Chikovore*: My formative social science training exposed me early to ‘critical’ conversations, including on the question of gender, and social processes and organization. My first job was as a research assistant on a large survey on male sexuality, relatively early in the HIV and AIDS pandemic. Participation in the survey was a huge learning moment; however, I would have noticed, from the exercise, the limitations of survey methodology, which did not allow participants room to elucidate thoughts, especially on a very sensitive subject. A few short stints later, I took up a graduate studentship, to understand the role of men in maternal mortality. Prevailing views then were that men had monolithic power and their behaviors/actions unidirectionally harmed women’s health; moreover, research on men largely used KAP (knowledge-attitude-perception) style survey-based methodologies. I approached the subject initially using qualitative methods (given the subject was little known); this approach revealed dynamics that challenged common expectations. From there, I became interested in men’s experience of gender power as a social construct, shaped in interaction with global, historical factors. This ‘intersectional’ lens has incrementally and in increasingly diversified ways informed my work, as I have straddled into equity, person-centered care, complexity, and related subjects.

*AD/JC*: While we have trained in diverse settings and spaces, over the years our shared interest in the social aspects of TB have led us to forge fruitful ties and contribute to a number of shared projects covering TB, stigma and mental health.

Our current research focus is Infectious disease, primarily TB and HIV, but also other conditions, harms and threats to wellbeing that arise out of the interactions of social stratifiers, structures, and movements. We apply a social science lens, drawing on disciplines of sociology and public health, largely, to study and address social determinants of illness and their intersections—how they intersect to create complex, compounded encounters (e.g. with health, wellbeing, disruption, resilience) and shape people’s lived experiences. In this our focus is on stigma as well as comorbid conditions or parallel social positions such as gender that could shape/potentiate stigma. We also focus on the construction and performance of gender as a social construct, which, in itself, can also potentiate stigma and mental strain through the pursuit of normative values and representations. This occurs at various levels, in different domains of social organization and processes, in different temporal and spatial sites. Our work thus addresses these two distinct yet intricately connected domains that influence, and equally are influenced by, social vulnerabilities and wellbeing, and health vulnerabilities and wellbeing, and the responses taken.

This means we lean heavily upon critical lenses that enable us to inquire how inequities arise, are sustained, and systematically come to bear on people’s risk of disease and engagement in health care and, in turn, how disease experience and meanings, engagement with healthcare, and systems put in place to manage these, can also accentuate inequities.

We carry a primary interest in naturalistic inquiry, and therefore qualitative approaches, because these allow for engagement with the complex, dynamic, inherently non-objective qualities (social underpinnings) of illness, and its social determinants—those which reflect a contextual construction such as stigma, gender and even the elusive character of co-occurring conditions that have an ingrained psychosocial basis such as mental health/illness. In line with principles of equity, inclusivity, and participation, qualitative methods allow for aiming and working towards centering contextualized subjectivities, voices, perceptions, and meanings of actors.

Along our journeys, we have increasingly engaged with the concept of equity as a core thread to looking at and seeking to help shift conversations and actions on health vulnerabilities and responses. This focal area in our work has also been propelled by growing mindfulness about important shortcomings in some of the approaches taken to health research and action. For one, the inequities that have defined and continue to define local and global health systems, structures, and processes have often not been given adequate attention. In addition, some of the actions and interventions have effectively reduced and simplified the complexity of behavior, meanings and experiences, and social processes and structures influencing them. More recently, we have witnessed the gaping fissures of inequity, and the role of structural factors in influencing vulnerability among global and local communities and groups, unmistakably visible in the wake of the current pandemic.

## Q2. What are the main problems that need addressing in the field?

*AD/JC*: Infectious diseases are often conceptualized in terms of defined borders—that is, as caused by specific agents, and spread through specific mechanisms, leading to specific symptoms, and hence requiring targeted intervention, with largely predictable prognostic and epidemiological outcomes. Those interventions tend to be bactericidal or virucidal-biomedical in nature. In addition, behavioral risk and responses of individuals or groups have been ‘objectively’ evaluated using standardized questionnaire techniques. The biomedical interventions and surveys provide critical tools and resources to apply in assessing and mitigating exposures, progression to illness, disease burden, and mortality. They are therefore all necessary, but not enough. There are ample determinants and impacts that bear on the risk, emergence, detection, treatment approaches, healthcare access/utilization, and outcomes of infection, and that are still elusive to those of us who work in this field, who seek to eliminate infection and mitigate impact. Notably absent is research into infection-related stigma. By the very nature of their transmissibility, infections are inherently feared and fear breeds ill-founded speculation and suspicion, separation, othering, distance and stigma. Equally, these various dimensions to illness and illness response and management (including stigma) lend themselves to how gender, and other identifiers intersect among each other, and across different levels or social processes, structural organization, contexts, and temporal moments. It is this complexity that, while now widely and routinely acknowledged as important, nevertheless continues to draw what seem to be piecemeal and measured responses. This is a key area for public health attention; specifically, efforts to design and facilitate person-centered and equitable management and prevention tools for people, families and communities affected by infectious diseases, and making requisite use of mixed-method approaches.

Whereas the infectious disease burden weighs more heavily towards countries of so-termed lower- and middle-income status (LMICs), and it is essential that the form stigma and mental health take in various settings in these countries is mapped well, there is need to balance this focus with an awareness that these issues present too in higher income settings, and affect disproportionately groups already marginalized in these contexts.

For infectious diseases, associated stigma prevents healthcare engagement, drives concealment of illness, and consequently encumbers the ability to receive crucial social and psychological support. The form and manifestation of stigma are similarly dependent on meanings assigned to being (seen as) sick with, or (being seen as) having acquired, a condition. Instances exist where having a disease is considered a sign of having breached a moral code, or reinforces associations made between being diseased and belonging to or having connection to a prejudiced group. Where a condition is publicly understood to be severe, or when a person shows severe symptoms suggestive of a known or feared disease, this also drives stigma and, in turn, can affect care seeking behavior and mental coping. Likewise, where a disease is associated with high likelihood of death or severe incapacitation, acquiring or suggesting one has the disease may imply physical weakness or some form of inferiority or difference. What is critical is that stigma and mental health of infectious disease are frequently shaped by contexts in which meanings given to the condition arise, and are reproduced and sustained. The contexts entail intersections of various identifiers including gender, race, social status, education, migrancy, ability/disability, age, among each other and within certain geographical and temporal sites. Being context-dependent necessitates that the forms stigma and mental health of infectious disease take in various contexts be elaborated, and with the use of appropriate methodologies and conceptual frameworks.

## Q3. How do you expect this field to develop in the next few years?

*AD/JC*: In addressing infectious disease, we (researchers) need to comprehensively characterize and invest fully in addressing the stigma they attract, and the mental anguish and in some cases serious mental health challenges they precipitate. Nowhere is this more critical than in the field of global health, where myriad other social inequities and constraints intervene on and bear upon infectious disease risk, emergence, prognoses, and impact.

We cannot reduce infectious disease to an agent or vector. The history and epidemiology of infectious diseases (consider the persistent numbers of people affected) shows us that this alone is insufficient. As research into the drivers and pathways of stigma and mental health emerge, there is huge potential to make more meaningful dents in infectious disease incidence, morbidity and mortality. Parallel to biomedical advancements, momentum has finally increased to address root social determinants of disease. We hope to see greater attention, resources and funds to support these dual efforts.

## Q4. What are you most excited about in your role as a guest editor for this collection?

*AD/JC*: We are excited that a publication powerhouse such as BMC and their new journal BMC Global and Public Health are vested in this topic; to invite the wide breadth of research enterprises focussed on this important topic, to see the depth in methods and the innovations being applied, and where and how this topic is being studied (different global settings, diversity of approaches, forms of knowledge translation and exchange), and to see this potential go-to resource for researchers starting to venture into these discussions, but also those who are already vested.

## Data Availability

Not applicable.

